# Higher Mast Cell Accumulation in Human Adipose Tissues Defines Clinically Favorable Obesity Sub-Phenotypes

**DOI:** 10.3390/cells9061508

**Published:** 2020-06-20

**Authors:** Nir Goldstein, Yarden Kezerle, Yftach Gepner, Yulia Haim, Tal Pecht, Roi Gazit, Vera Polischuk, Idit F. Liberty, Boris Kirshtein, Ruthy Shaco-Levy, Matthias Blüher, Assaf Rudich

**Affiliations:** 1 Department of Clinical Biochemistry and Pharmacology, Ben-Gurion University of the Negev, Beer-Sheva 8410501, Israel; goldsnir@tauex.tau.ac.il (N.G.); beckyu@bgu.ac.il (Y.H.); pacht@post.bgu.ac.il (T.P.); 2 Department of Epidemiology and Preventive Medicine, School of Public Health, Sackler Faculty of Medicine and Sylvan Adams Sports Institute Tel Aviv University, Tel-Aviv 6997801, Israel; gepner@tauex.tau.ac.il; 3 Institute of Pathology, Soroka University Medical Center Ben-Gurion University of the Negev, Beer-Sheva 84101, Israel; kezerley@bgu.ac.il (Y.K.); rsl@bgu.ac.il (R.S.-L.); 4 The Shraga Segal Department of Microbiology Immunology and Genetics Faculty of Health Sciences, Ben-Gurion University of the Negev, Beer-Sheva 8410501, Israel; gazitroi@bgu.ac.il; 5 The National Institute of Biotechnology in the Negev, Ben-Gurion University of the Negev, Beer-Sheva 8410501, Israel; 6 Soroka University Medical Center and Faculty of Health Sciences, Ben-Gurion University of the Negev, Beer-Sheva 84101, Israel; VeraP@clalit.org.il (V.P.); iliberty@bgu.ac.il (I.F.L.); borkirsh@bgu.ac.il (B.K.); 7 Department of Medicine, University of Leipzig, 04103 Leipzig, Germany; bluma@medizin.uni-leipzig.de; 8 Helmholtz Institute for Metabolic, Obesity and Vascular Research (HI-MAG) of the Helmholtz Zentrum München at the University of Leipzig and University Hospital Leipzig, 04103 Leipzig, Germany

**Keywords:** obesity, type 2 diabetes, bariatric surgery, adipose tissue, mast cells

## Abstract

The identification of human obesity sub-types may improve the clinical management of patients with obesity and uncover previously unrecognized obesity mechanisms. Here, we hypothesized that adipose tissue (AT) mast cells (MC) estimation could be a mark for human obesity sub-phenotyping beyond current clinical-based stratifications, both cross-sectionally and prospectively. We estimated MC accumulation using immunohistochemistry and gene expression in abdominal visceral AT (VAT) and subcutaneous (SAT) in a human cohort of 65 persons with obesity who underwent elective abdominal (mainly bariatric) surgery, and we validated key results in two clinically similar, independent cohorts (*n* = 33, *n* = 56). AT-MC were readily detectable by immunostaining for either c-kit or tryptase and by assessing the gene expression of KIT (KIT Proto-Oncogene, Receptor Tyrosine Kinase), TPSB2 (tryptase beta 2), and CMA1 (chymase 1). Participants were characterized as VAT-MC^low^ if the expression of both CMA1 and TPSB2 was below the median. Higher expressers of MC genes (MC^high^) were metabolically healthier (lower fasting glucose and glycated hemoglobin, with higher pancreatic beta cell reserve (HOMA-β), and lower triglycerides and alkaline-phosphatase) than people with low expression (MC^low^). Prospectively, higher MC accumulation in VAT or SAT obtained during surgery predicted greater postoperative weight-loss response to bariatric surgery. Jointly, high AT-MC accumulation may be used to clinically define obesity sub-phenotypes, which are associated with a “healthier” cardiometabolic risk profile and a better weight-loss response to bariatric surgery.

## 1. Introduction

The inflammation of adipose tissue (AT) may link obesity to its cardiometabolic comorbidities. Although macrophages (CD68+ cells) were the first immune cell type realized to infiltrate adipose tissue (AT) in obesity and to associate with obesity-related metabolic dysfunction [1], the current view in the field engages virtually all immune cell types in obesity-associated AT inflammation, including T and B lymphocytes (and their sub-classes), dendritic cells, neutrophils, and natural killer (NK) cells [2]. Within the changing environment of adipose tissues in obesity, these cell types undergo complex phenotypic alterations, rendering them highly diverse. This challenges the efforts to establish clear contributions of specific cell types to obesity-associated metabolic deterioration, even for adipose tissue macrophages [3] (also recently reviewed in [4,5]). In 2009, elevated numbers of mast cells (MC) were demonstrated in obese AT, located mainly near micro-vessels, both in humans and in mouse models [6]. Mast cells are hematopoietic, bone-marrow-derived immune cells, which mature and differentiate in the tissue in which they eventually reside. Although mainly found in tissues with greater interaction with the outer environment such as the reticular layers of the skin, gastrointestinal system, and the airways, MC can be found in all organs and tissues. In AT of lean mice, MC are more abundant in subcutaneous AT (SAT) than in epididymal, mesenteric, or perirenal fat pads. Interestingly, obesity is associated with increases in MC numbers in all white AT depots besides SAT [7].

The physiological/functional impact of increased numbers of AT-MC in obesity currently remains controversial: Two independent groups demonstrated that MC-deficient mice by mutation in the growth factor receptor KIT (KitW-sh/W-sh), which is required for MC development, are protected from diet-induced obesity and its co-morbidities [6,8]. In contrast, two different, none KIT-dependent MC-deficient mouse models (Cpa3cre/+ (Cre recombinase in the carboxypeptidase A locus) and Mcpt5-Cre+ R-DTA+ (MC specific expression of diphtheria toxin A)) exhibited no phenotypic impact on the development of obesity or on its metabolic consequences, including insulin resistance, hepatic steatosis, or inflammation [9,10].

In humans, the AT of people with obesity exhibited higher MC numbers compared to lean patients, both in SAT [6,11] and VAT [11]. Importantly, this finding was particularly evident among patients with obesity and type 2 diabetes [11], suggesting a link between high AT-MC infiltration and the severity of obesity-related metabolic disturbance. Consistently, a sub-analysis among 20 persons with obesity suggested that higher MC numbers associate with higher fasting glucose and HbA1c [11]. Interestingly, in that study, two sub-populations of AT-MC were assessed based on the expression of their proteases—tryptase+ and tryptase+/chymase+ MC (MCT and MCTC, respectively), rendering tryptase, the gene product of tryptase beta-2 (TPSB2), a common marker for MC. Yet, the ratio between these two sub-populations remained similar in different depots and in leanness versus obesity. Isolated MC from the SAT of patients with obesity and type 2 diabetes were more activated, releasing more inflammatory cytokines and proteases such as tryptase [11]. Correspondingly, serum tryptase concentrations were higher in people with obesity compared to lean [6]. In a different study examining a mixed cohort of persons without or with obesity, the expression of MC-tryptase (TPSB2) in VAT did not associate with parameters of glucose homeostasis or insulin sensitivity [12]. Collectively, obesity seems to associate with increased numbers of MC in AT. Mouse models so far yielded conflicting results. Human studies suggest that higher AT-MC numbers (i.e., accumulation), and perhaps their activation, may associate with poor glycemic control, but assessment of whether and how AT-MC associate within cohorts of patients with obesity, and whether it corresponds to obesity sub-phenotypes, is limited.

In the present study, we aimed to address this current gap of knowledge by hypothesizing that among persons with obesity, higher numbers of AT-MC associate with an obesity phenotype characterized by a poorer metabolic profile. We sought to assess the relevance of such MC-based obesity sup-phenotyping both in cross-sectional analyses of patients with obesity, with and without type 2 diabetes, and for predicting patients’ response to bariatric surgery.

## 2. Materials and Methods

### 2.1. Human Cohorts

We recruited persons with obesity (Body mass index (BMI) ≥ 30 kg/m^2^) undergoing elective abdominal surgery (mainly bariatric surgery or elective cholecystectomy) as part of the coordinated human adipose tissue bio-banks in Beer-Sheva, Israel (*n* = 65, main cohort) and in Leipzig, Germany (*n* = 32 and *n* = 56, validation cohorts 1 and 2, respectively) (Table 1). Prior to operation, under overnight fasting conditions, body weight and blood samples were obtained. Both visceral (omental) and superficial-subcutaneous adipose tissues biopsies were obtained during the surgery and processed for histology and gene expression using coordinated methodologies, as we previously described in detail [13,14]. Persons were identified as normoglycemic if fasting plasma glucose (FPG) levels were lower than 5.6 mmol/L, HbA1c ≤ 38 mmol/mol (5.6%), and with no anti-diabetic medications on the day of operation. Prediabetes was defined as FPG 5.6–6.9 mmol/L and HbA1c 39–46 mmol/mol (5.7–6.4%), and type 2 diabetes if glucose ≥ 7.0 mmol/L or HbA1c ≥ 48 mmol/mol (6.5%). In 13 patients (20%), in whom glycemic status was ambivalent, medical records were screened up to 4 months pre-operation for additional FPG and HbA1c measurements, and final categorization was made by co-author IFL, who is a Diabetologist. A homeostatic model assessment of insulin resistance (HOMA-IR) and homeostatic model assessment of beta cells reserve (HOMA-β) were calculated as follows: HOMA-IR: (FPG (mmol/L) × Insulin (µIU/mL)/22.5) and HOMA-β: (20 × Insulin (µIU/mL)/ FPG (mmol/L)-3.5) [15]. For post-operation follow-up sub-study, only people undergoing bariatric surgery for the first time and for whom postoperative information was available were included. In addition to the main Beer-Sheva cohort, we included two independent cohorts—validation cohorts 1 and 2, with *n* = 32 and *n* = 56 individuals, respectively, all with obesity (BMI range: 30–75 kg/m^2^, Table 1), from the University of Leipzig Obesity Treatment Center. Paired abdominal subcutaneous and omental adipose tissue biopsies were taken during elective sleeve gastrectomy, Roux-en-Y gastric bypass, hernia, or cholecystectomy surgeries and processed as previously described [16]. As for validation cohort 2, we included data from 56 patients who underwent a two-step bariatric surgery strategy with laparoscopic gastric sleeve resection as the first step and a Roux-en-Y gastric bypass as the second step 12 ± 2 months later (Table 1). At both time points, serum/plasma samples, omental, and abdominal subcutaneous adipose tissue biopsies were obtained. All patients provided before the study a written informed consent to participate, and all procedures were approved in advance by the local ethical committees and conducted in accordance with the declaration of Helsinki guidelines (0348-15-SOR; for Leipzig cohorts:, 017-12-23012012, and Reg. No. 031-2006).

### 2.2. RNA Extraction and Quantification

mRNA was extracted as described previously [13]. Briefly, 300 mg of tissue were minced and extraction was done with an RNeasy lipid tissue minikit (Qiagen. Hilden Germany). cDNA was produced using the reverse transcriptase kit (Applied Biosystems. Beverly, MA, USA). mRNA was quantified using the Taqman system, where expression levels of selected genes were calculated as a fold from AT-specific endogenous control genes (PGK1 and PPIA) [17] and calculated as 2^−∆∆ct^ [18]. Probes assay IDs are in Table S1. For the Leipzig validation cohort 1, mRNA expression of candidate genes (cKIT, CMA1, TPSB2) was analyzed using Illumina human HT-12 expression chips. RNA integrity and concentration were examined using an Agilent 2100 Bioanalyzer (Agilent Technologies, USA). In validation cohort 2, additional measurements of cytokine serum concentrations and adipose tissue expression (Interleukin -6 (IL-6), Interleukin -1beta (IL-1beta), Tumor necrosis factor alpha (TNFalpha)) were performed as described previously [3].

### 2.3. Histology and Immunostaining

Immunostaining for C-Kit (Dako (Agilent), Santa Clara, CA USA) or Tryptase (ThermoFisher. Waltham, MA USA), and CD68 (Dako (Agilent), performed in a sub-cohort of *n* = 30 from the Beer-Sheva cohort), is performed routinely in clinical pathology to stain tissue MC and macrophages, respectively. Immunostaining was performed in 5 microns-thick sections from paraffin embedded visceral AT (VAT) and subcutaneous (SAT) samples as described before [14]. Cell count/field was performed, blindly and independently, by two pathologists (Y.K and R.S-L) using an Olympus BX43 light microscope, in 10 consecutive high-power fields (X400), and the number of C-Kit+ and tryptase+ per 100 adipocytes was calculated.

### 2.4. Statistical Analyses

Baseline clinical characteristics values are presented as mean ± SD. An independent t-test was used to compare between groups with Levene’s test for equality of variances. In cases of non-normal distribution, ln-transformation was made. Comparison between percentages of medications usage was calculated with a chi-square test. An ANOVA test was used to compare between the four groups when sex, age, and diabetes stratification was used in combination with MC high/low stratifications. In order to detect differences between the four groups, Least Significant Difference (LSD) post-hoc analysis was used. A 2 × 2 ANOVA was performed to test the relationship between non- and type 2 diabetes groups and the MC group (MClow/high) using an age-adjusted model. Extreme outliers were excluded using the interquartile range 3 × (Q3-Q1). We also used Spearman’s correlation to test the association between c-Kit+, tryptase+, and CD68^+^ cells, clinical parameters, and MC-related genes. A linear regression model was used to assess the association between VAT-CMA1 high/low expression and percentage of weight loss. The model was adjusted for baseline BMI or surgery type. Analyses were performed using SPSS Version 20.0 (SPSS, Inc., Chicago, IL, USA) and GraphPad prism (8.4.2., San-Diego, CA, USA). All tests were two-tailed and *p* < 0.05 was considered statistically significant.

## 3. Results

### 3.1. Assessing AT-MC Histologically and by Gene Expression

To test if the degree of AT-MC accumulation may reflect obesity sub-phenotypes, we first investigated a cohort of persons with obesity (BMI ≥ 30 kg/m^2^, *n* = 65) (Table 1, Beer–Sheva cohort). Among the persons with obesity, 38 persons (58.5%) were morbidly obese (Class III, BMI ≥ 40 kg/m^2^). Compared to those with 30 ≤ BMI < 40 kg/m^2^, persons with morbid obesity differed only in weight, BMI, waist circumference, and low-density lipoprotein (LDL).

**Figure 1:**
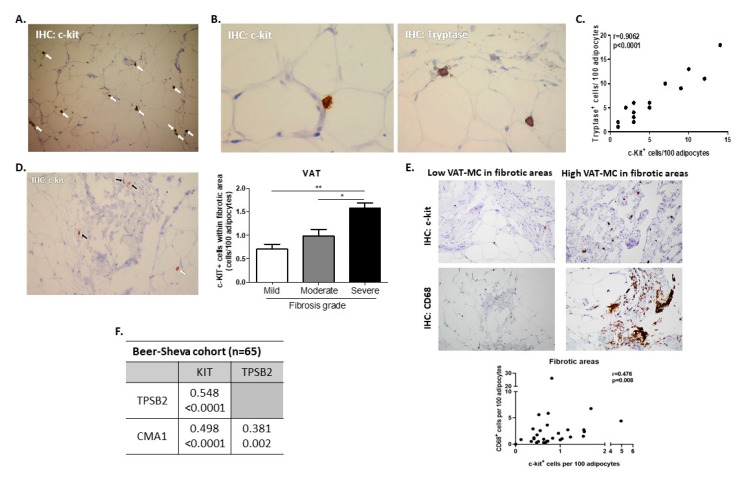
Identification of human visceral (omental) adipose tissue mast cells (AT MC). AT stained for c-KIT Proto-Oncogene Receptor Tyrosine Kinase (C-Kit+, white arrows) with (**A**) ×100 and (**B**) ×400 magnification (left), or stained for tryptase+ cells ×400 (**B**, right). (**C**) Spearman’s correlation between %C-Kit+ and %tryptase+ cells (percentage being per 100 adipocytes) in serial AT sections (*n* = 14). (**D**) C-Kit+ is discernable both within fibrotic areas (black arrows) and around adipocytes (white arrow), and bar graph—the number of C-Kit+ cells in sections rated by clinical pathologists (co-authors YD and RSL) as exhibiting mild, moderate, or severe degree of fibrosis. (**E**) Representative histological sections from two representative patients—one with low and the second with high MC in fibrotic areas within VAT, stained for either C-Kit (MC) or CD68 (macrophages). Graph below depicts Spearman’s correlation between %C-Kit+ and %CD68+ cells (i.e., macrophages, percentage being per 100 adipocytes) in serial VAT sections within the fibrotic areas, *n* = 30. (**F**) Spearman’s intercorrelations (*n* = 65) between VAT MC-related genes (KIT, TPSB2, and CMA1). In each correlation, the upper line indicates *r*(ρ) value and lower line—*p*-value.

We contemplated both histological and gene expression approaches to estimate AT-MC accumulation, utilizing several genes/proteins considered as MC-specific, including KIT/c-Kit (c-KIT Proto-Oncogene Receptor Tyrosine Kinase), TPSB2/Tryptase (mast cell tryptase beta II), and CMA1 (mast cell chymase 1 [19]). The specificity of selected genes to MC is presented in Supplemental Figures S1–S3. By immunohistochemistry, c-Kit+ or tryptase+ cells were readily discernable in human visceral AT from obese patients (Figure 1A,B). When assessing serial sections, the number of c-Kit+ cells per 100 adipocytes (percentage of c-Kit+) highly correlated with the percentage of tryptase+ cells (Figure 1C). AT-MC could be detected both in fibrotic areas within AT and dispersed between the adipocytes, being more prevalent in VAT sections rated by Clinical Pathologists (co-authors YK and RSL) as exhibiting more severe fibrosis (Figure 1D, for SAT see Figure S4). Interestingly, AT-MC correlated with CD68^+^ macrophages only within fibrotic areas of the tissues (Figure 1E), but not in parenchymal areas between adipocytes (*r*(ρ) = 0.264, *p* = 0.159, *n* = 30). Using a gene expression approach, the expression of all three MC genes was significantly intercorrelated in both VAT (Figure 1F) and SAT (not shown). Moreover, VAT-KIT gene expression levels correlated with visceral percentage of c-Kit+ cells (log-transformed, *r* = 0.363, *p* = 0.038), and VAT-CMA1 expression correlated with both percentage of c-Kit+ and percentage of tryptase+ (*r*(ρ) = 0.348, *p* = 0.044; *r*(ρ) = 0.561, *p* = 0.008, respectively). Consistent with the proposed association between MC and fibrosis, we found significant associations between AT-MC genes and the expression of collagens 1A1, 3A1, and 6A1 in VAT (Table 2), but less so in SAT that exhibited only correlations between SAT-MC genes with collagen 6A1 (Table S3).

In the independent validation, cohort 1 of *n* = 32 patients with obesity whose VAT gene expression was assessed by microarrays [16], all MC genes probe sets (KIT, TPSB2, CMA1) exhibited significant negative correlations with macrophage gene probes (CD68, Mac2, IGTAX(CD11c)). Most significant were correlations between higher VAT-KIT and lower expression of CD68 (*r*(ρ) = −0.559, *p* = 0.001, *n* = 32), and between higher VAT-CMA1 and lower expression of Mac2 (*r*(ρ) = −0.448, *p* = 0.010, *n* = 32). Consistently, AT-MC genes’ expression in VAT negatively associated with the number of macrophage crown-like structures (CLS) in validation cohort 2 (*r*(ρ) = −0.294, *p* = 0.047, *n* = 56). Jointly, these analyses demonstrate (1) that AT-MC accumulation can be estimated confidently by histology and gene expression, (2) that histologically, AT-MC may correlate with the abundance of macrophages only within fibrotic regions. By gene expression, AT-MC do not positively correlate, and may even exhibit a negative association with the total abundance of AT macrophages.

### 3.2. Cross-Sectional Analyses

Next, we wished to challenge our hypothesis that higher AT-MC accumulation would signify an obese sub-phenotype characterized by worse cardiometabolic risk. For this purpose, we used the larger Beer-Sheva cohort of *n* = 65 patients with obesity to compare those with obesity and high versus low VAT-MC accumulation. Given the suspicion that KIT may not be sufficiently MC-specific [9], but that c-Kit and TPSB2 largely identify the same cells in the tissue (Figure 1D), and that AT-MC express TPSB2−/+ CMA1 [11], we based MC gene expression stratification on the combined VAT expression levels of TPSB2 and CMA1: Patients were categorized as VAT-MC^low^ only if the AT expression of both TPSB2 and CMA1 genes was below the median level of expression in the entire cohort, whereas VAT- MC^high^ were all those with above-median gene expression in either or both genes.

**Figure 2:**
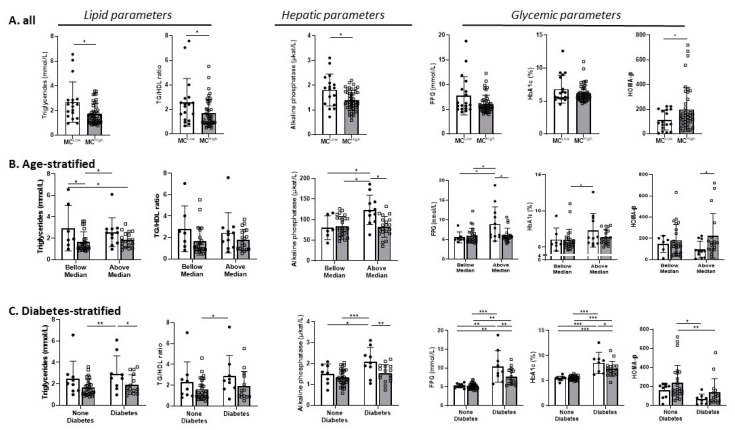
Comparison of clinical parameters between participants with high versus low MC accumulation in VAT. MC^low^ (white bars) were defined as people in whom the expression of both *TPSB2* and *CMA1* were below the median value of the cohort for each of the genes; all others were defined as MC^high^ (i.e., with either one or two of the genes expressed above median, gray bars). (**A**) Differences in triglycerides (TG)/HDL ratio, alkaline phosphatase, FPG, Hemoglobin A1c (HbA1c), and HOMA-β between VAT- MC^low^ versus VAT- MC^high^. (**B**,**C**) Same analysis as in **A**, but the cohort was stratified by age (below/above median = 45 years, B), or type 2 diabetes status (**C**). Age-adjusted 2 by 2 ANOVA was used to test for variance. Age-adjusted Least Significant Difference (LSD) post-hoc test was used to compare between groups. Values are expressed as mean ± SD. * *p* < 0.05, ** *p* < 0.01, *** *p* < 0.001.

Contrary to our hypothesis, patients with obesity and low VAT-MC gene expression (below median expression of both TPSB2 and CMA1) were either not significantly different, or in some parameters trended to, or exhibited significantly worse clinical parameters (Table 1, Figure 2A). Significant differences between patients with obesity and VAT-MC^low^ and VAT-MC^high^ were observed in triglyceride (TG) levels and HDL (high-density lipoprotein) ratio, in circulating alkaline phosphatase and in HOMA-β. Sensitivity analysis in which outliers were excluded rendered the difference in HOMA-β between those with VAT-MC^low^ versus VAT-MC^high^ insignificant. We could not attribute the differences to the different use of medications (Table S4). Validation cohorts 1 and 2 exhibited similar trends to those observed in the main cohort (Table 1), and such trends were less robust when defining VAT-MC accumulation based on the expression of KIT alone (Table S2). To further explore if this unexpected association between higher VAT-MC expression and seemingly improved clinical markers of cardiometabolic risk are contributed by specific sub-groups of patients, the analysis was repeated after stratification of the main cohort by either sex, age (above/below median of 45 years old), T2DM status, and obesity class (Figure 2 and Figure S5, respectively). Remarkably, significant differences exhibiting improved parameters in VAT-MC^high^ were more readily observed in females (Figure S5A), in participants whose age was above median (Figure 2B), in patients with type 2 diabetes mellitus (T2DM) (Figure 2C), and in those with BMI 30.0–39.9 kg/m^2^ (Figure S5B). Intriguingly, a significant *p* interaction was observed between diabetes status and VAT-MC in FPG and in HbA1c (*p* = 0.016 and *p* = 0.041, respectively). This could not be attributed to different diabetes duration or different use of medications (Tables S4 and S5).

**Figure 3:**
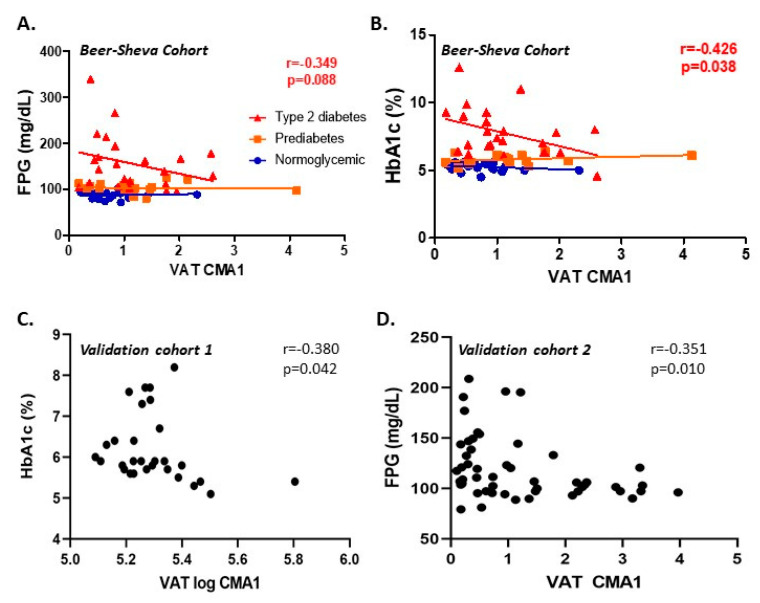
Associations between VAT CMA1 gene expression and glycemic parameters in the Beer–Sheva and Leipzig independent cohorts. Spearman’s associations between VAT CMA1 gene expression and FPG (**A**) or HbA1c (**B**) among the Beer–Sheva cohort (*n* = 65, all with body mass index ≥ 30 kg/m^2^), stratified into those with normal glucose homeostasis (normoglycemic, blue circle), prediabetes (orange square), or type 2 diabetes (red triangle). *r* denotes Spearman’s rank correlation coefficient (Rho, ρ) among participants with type 2 diabetes. (**C**) Spearman’s rank correlation between log-transformed CMA1 expression in VAT assessed by microarray and HbA1c in the Leipzig validation cohort 1 (*n* = 32, all with BMI ≥ 30 kg/m^2^). (**D**) Spearman’s rank correlation between CMA1 expression in VAT and FPG in Leipzig validation cohort 2 (*n* = 56, all with BMI ≥ 30 kg/m^2^).

Indeed, by correlation (rather than group statistics) analysis, a significant negative association was observed between VAT-CMA1 expression level and FPG or HbA1c among the sub-group of patients with obesity and T2DM (association with TPSB2 exhibited a similar trend) (Figure 3A,B, respectively). Importantly, this association was also evident in the two independent Leipzig cohorts (Figure 3C,D). In SAT, associations between the expression of MC genes and clinical parameters in the 3 cohorts is presented in Table S6, displaying less consistent cross-sectional associations than those observed with VAT-MC. Jointly, these cross-sectional analyses disproved our initial hypothesis that increased VAT-MC accumulation would signify a worse obesity sub-phenotype. Rather. they provided evidence that the reverse association may in fact hold true. This was particularly apparent in certain sub-groups of patients with obesity, such as females, older patients, in those whose obesity was also complicated with T2DM, and in patients with obesity class I + II.

### 3.3. In Vitro Analyses

**Figure 4:**
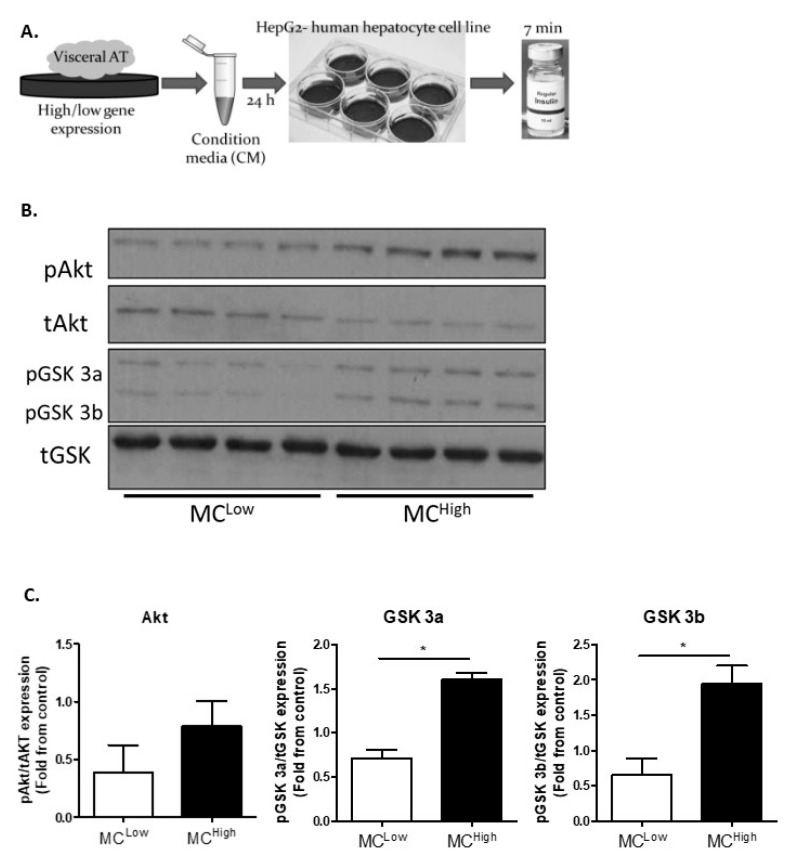
Insulin signaling in HepG2 cells after treatment with condition media from the VAT of obese MC^low/high^. (**A**) Schematic experimental design: VAT explants were incubated in media for 24 h for the preparation of conditioned media (CM). CM was used to expose human hepatocyte cell line (HepG2) for 24 h, followed by insulin (100 nM) stimulation for 7 min. (**B**) Representative blots of Western blot analysis for the following antibodies: p(Ser473)Akt, tAkt, p(Ser21/9)GSK3, and tGSK3. (**C**) Expression quantification of the proteins above (**B**) from two independent experiments, *n* = 4 for each group. Mann–Whitney non-parametric test was performed in order to compare means. Values are expressed as mean ± standard error of the mean (SEM), * *p* < 0.05.

The results described so far suggest that increased VAT-MC infiltration may cross-sectionally associate with a more favorable obese sub-phenotype, but the underlying mechanism remains obscure. To this end, given the centrality of the VAT–liver access in metabolic health/dysfunction in obesity, particularly when complicated with diabetes, we hypothesized that secreted factors from VAT-MC^high^ AT mediate a more metabolically favorable communication with liver cells compared to VAT-MC^low^ AT. To test this hypothesis, we treated human hepatocyte-derived cells with conditioned media (CM) obtained from the VAT of obese people with either high or low MC gene expression and assessed the ensuing acute signaling response to insulin stimulation (Figure 4A). HepG2 cells treated with CM from VAT-MC^high^ were more insulin-responsive compared to cells treated with CM from VAT-MC^low^, as determined by the insulin-induced phosphorylation level of GSK3 (Figure 4B,C).

### 3.4. Prospective Analyses

**Figure 5:**
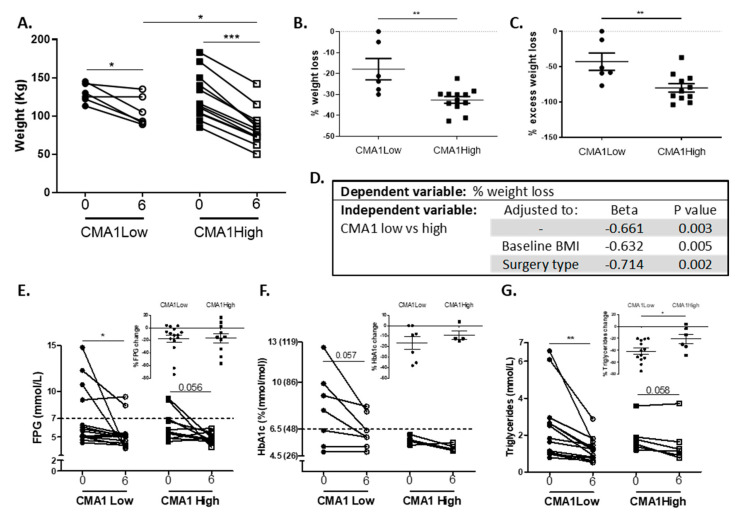
Six months postoperative weight reduction and metabolic changes comparison between people with VAT CMA1 high or CMA1 low. (**A**) Body weight change between operation day (0) and 6 months post-surgery in persons with low expression (i.e., below median) of CMA1 (*n* = 6, circles) or high CMA1 mRNA levels (n = 12, squares). Percentage of weight loss (**B**) from preoperative weight and of excess weight loss six months following bariatric surgery (**C**). A paired t-test was used to compare between pre-operation (0) and 6 months post-surgery (6). A Mann–Whitney non-parametric test was performed in order to compare means between CMA1 low and CMA1 high. * *p* < 0.05, ** *p* < 0.01. (**D**) Multi-variant model for association between VAT CMA1 groups and 6 months weight changes as dependent variable, adjusted to baseline BMI and surgery type. Change in FPG (**E**), HbA1c (**F**), and triglycerides (**G**) between operation day (0) and 6 months post-surgery in persons with low expression CMA1 or high CMA1 mRNA levels. The percentage of change in FPG, HbA1c, and triglycerides (insets). Values are expressed as mean ± standard error of the mean (SEM).

Beyond the potential to utilize the estimation of MC accumulation of VAT for sub-typing obese patients in a cross-sectional analysis, we aimed to determine in a subset of patients from the main cohort, for whom post-bariatric surgery data was already available, whether the expression level of MC genes in VAT can predict clinically meaningful outcomes of intervention. Using the same approach to assess the VAT-MC accumulation level based on both TPSB2 and CMA1, patients with VAT-MC^high^ (above median value of the entire cohort) lost significantly more weight 6 months after bariatric surgery compared to VAT-MC^low^ (31.7 ± 1.6% versus 13.4 ± 6.5% of baseline body weight, corresponding to 79.1 ± 5.7% and 30.7 ± 14.5% loss of excess body weight, respectively, *p* = 0.002). For this analysis, we found a yet stronger association when using VAT-CMA1 expression only (Figure 5A,B): People with obesity and high VAT-CMA1 expression achieved 1.74-fold greater weight reduction compared to obese people with low VAT-CMA1 expression 6 months post-surgery (*p* = 0.016). This difference remained significant (*p* = 0.038) even when the two patients that showed minimal weight loss were excluded. Different weight losses corresponded to a 1.9-fold greater loss of excess body weight among the VAT-CMA1^High^ compared to the VAT-CMA1^Low^, *p* = 0.008, Figure 5C). The difference in weight reduction between the two groups remained significant even when adjusting to either baseline BMI or surgery type (Figure 5D). Intriguingly, people with obesity and low VAT-CMA1 had higher levels of FPG (Figure 5E), HbA1c (Figure 5F), and triglycerides (Figure 5G) pre-operation, and despite lower extent of weight loss, exhibited a greater reduction, particularly in triglycerides, 6 months postoperatively. Yet, unlike with the weight change, these differences could be explained by their higher baseline values (data not shown). We did not observe any additional difference in the other clinical parameters that reached statistical significance (data not shown).

To strengthen the limited observation achievable with our main cohort, we utilized validation cohort 2 of the Leipzig bio-bank, in which 1 year post-laparoscopic sleeve gastrectomy data was available (see details of this 2-step bariatric surgery cohort in the Methods section). As expected, there were significant declines in weight and anthropometric measures, in glycemic and insulin resistance indices, and in lipids (increase in HDL) (Table 3).

**Figure 6:**
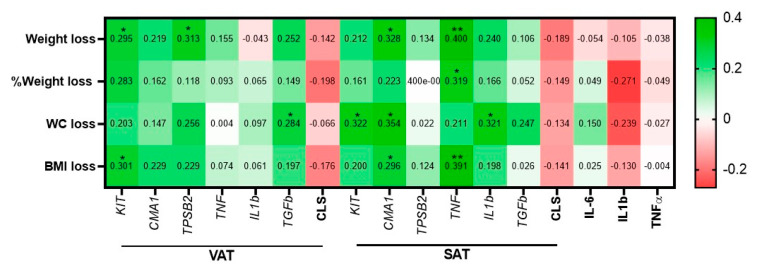
Correlation between baseline parameters and 1 year postoperative weight loss response in validation cohort 2. Values are *r*(ρ) of Spearman’s correlations. WC: waist circumference. * *p* < 0.05; ** *p* < 0.01.

Among circulating cytokines tested, IL-6 robustly declined (*p* < 0.0001), and a significant reduction was observed in CRP levels (*p* = 0.024). Several inflammatory markers were measured in both VAT and SAT. In VAT, both macrophage CLS number and IL1b expression decreased significantly (*p* < 0.0001 and *p* = 0.003, respectively). Among MC genes, only CMA1 expression exhibited a significant expression reduction (delta of 0.3 ± 1.0, *p* = 0.044). Similar results were achieved in SAT, with significant reductions in macrophages CLS and IL1b (*p* = 0.005, *p* = 0.035, respectively), but with no significant change in CMA1 expression but with a reduction in TGFbeta expression (*p* = 0.024). Next, we looked for associations between baseline VAT and SAT MC genes expression and selected inflammatory markers and changes in anthropometric parameters 1 year following bariatric surgery (Figure 6). In this cohort of extremely obese patients (average: BMI > 50 kg/m^2^, Table 1), the baseline expression of KIT and TPSB2 in VAT seemed to be better predictors of greater weight loss 1 y postoperatively, reaching statistically significant associations (*r*(ρ) = 0.295, *p* = 0.044, *r*(ρ) = 0.313, *p* = 0.03, respectively). Baseline VAT-KIT was also associated with BMI loss (*p* = 0.04) and trended with percentage of weight loss (*r*(ρ) = 0.283, *p* = 0.054). This is somewhat consistent with the results of the main cohort of less obese patients (Table 1); baseline VAT-CMA1 in validation cohort 2 trended to associate with a greater loss in BMI 1 y after surgery (*r*(ρ) = 0.229, *p* = 0.099). In addition, baseline VAT-TGFbeta was also associated with a greater reduction in WC 1 year postoperatively (*r*(ρ) = 0.284, *p* = 0.048).

Interestingly, unlike the cross-sectional analyses (Section 3.2), in which VAT-MC exhibited more robust associations with patients’ clinical characteristics than SAT-MC (Figure 3 and Figure S5 and Table S6), SAT-MC genes and inflammatory markers exhibited more associations with 1 year postoperative outcomes (Figure 6). Higher SAT-CMA1 associated with greater weight, waist circumference (WC), and BMI loss (*r*(ρ) = 0.328, *p* = 0.015, *r*(ρ) = 0.354, *p* = 0.015 and *r*(ρ) = 0.296, *p* = 0.030, respectively). Baseline SAT-KIT similarly associated with greater WC loss (*p* = 0.045). Intriguingly, higher SAT-TNFalpha expression highly associated with greater weight, percentage weight, and BMI losses (*r*(ρ) = 0.400, *p* = 0.004, *r*(ρ) = 0.319, *p* = 0.024 and *r*(ρ) = 0.391, *p* = 0.005, respectively), as did a higher baseline expression of SAT-IL1beta (association with WC decline—*r*(ρ) = 0.321, *p* = 0.028). Baseline macrophages CLS numbers, in both VAT and SAT, and circulating cytokines were not significantly associated with a reduction in any anthropometric parameters. Moreover, although non-significantly, they exhibit negative *r*(ρ) coefficients with 1 year postoperative weight loss measures, which is opposite to the associations (both statistically significant or non-significant) between higher baseline VAT or SAT-MC gene expression and greater weight loss following surgery.

Jointly, these results provide proof-of-principle for VAT and SAT MC and inflammatory gene expression as putative molecular predictors of weight loss and metabolic improvement six months to 1 y following bariatric surgery.

## 4. Discussion

In this study, we hypothesized that AT-MC accumulation may aid in characterizing obesity sub-phenotypes, and we addressed this hypothesis by cross-sectional and prospective/predictive analyses of three independent cohorts of patients, all with obesity. Our results suggest the following: (1) We anticipated that greater AT-MC accumulation of VAT would characterize a more adverse obese phenotype as determined by cardiometabolic risk parameters. This hypothesis could be clearly rejected by the data. Moreover, contrasting our initial hypothesis, we found that MC numbers and MC-related gene expression in omental fat (VAT) associated in 3 independent cohorts with a better cardiometabolic risk profile, associations that are not consistently observed with SAT. Conducting post-hoc exploratory analyses, this association was strengthened when stratifying the larger cohort by basic clinical sub-groups, such as sex, age, and type 2 diabetes status. In the latter group, high VAT-MC gene expression associated with a metabolically healthier phenotype compared to those with low MC gene expression pattern. In some parameters, patients with obesity and diabetes, and with high MC gene expression in VAT were indistinguishable from patients with obesity without diabetes. (2) In vitro studies suggest that VAT with high MC gene expression communicates more favorably with liver-derived cells, rendering them more insulin-responsive compared to cells exposed to VAT with low MC gene expression. (3) We observed that analyzing VAT-MC gene expression in samples obtained during bariatric surgery can provide predictive information on the degree of weight loss and metabolic response to bariatric surgery: high pre-operative VAT CMA1 or KIT expression predicted significantly greater weight loss 6 months or 1 year postoperatively, respectively. Unlike in the cross-sectional analyses, higher SAT-MC gene expression also correlated with greater weight-loss response to bariatric surgery.

Whether AT MC accumulation plays a role in obesity is still debatable. In mice, results are contradictory depending on the models used to generate MC deficiency [6,8,9,10]. Moreover, MC depletion via c-kit prevented obesity development [6,8,9,10], and therefore it may not contribute to uncovering MC’s role when obesity is established, and how it relates to the development of obesity-associated cardiometabolic morbidity. In humans, particular interest in obesity sub-typing/sub-phenotyping is emerging [20], since with its high prevalence, there is a pressing need to increase the identification of obesity subtypes that may require more intensive treatment. Compared to lean people, persons with obesity exhibit higher numbers of MC both in VAT and SAT [6,11], especially if obesity was accompanied with type 2 diabetes mellitus [11]. Compared to the study by Divoux et al. that examined persons with obesity with/without type 2 diabetes (albeit with a limited *n* = 10 in each group) [11], we could not detect a clear increase in MC parameters in VAT from persons with diabetes compared to those without (*n* = 40 and 25, respectively). Our findings suggest that a larger cohort than that previously analyzed may have been required to uncover heterogeneity within the group of patients with obesity and type 2 diabetes. Indeed, associations between VAT-MC and metabolic parameters were mainly discernable among those with diabetes and higher expression levels of MC genes in VAT. In those patients, VAT-MC gene expression was associated with a healthier metabolic phenotype, suggesting a possible compensatory increase in VAT-MC in response to metabolic impairment, which, differently than previously suspected, exerts protective effects on metabolic health.

This work has some limitations. Our main cohort’s size (*n* = 65), though the largest (to our knowledge) used to examine in humans AT-MC, is still relatively small, and the sub-analysis for predicting response to surgery includes an even smaller sub-cohort (*n* = 18). In addition, the high HOMA-β values may indicate the possibility that we investigated a specific obesity sub-group with relatively high beta cell reserve. Yet, we confirmed the key findings in 2 independent cohorts, using different approaches to estimate AT-MC gene expression. Findings were opposite to our pre-defined hypothesis that increased MC accumulation would associate with a worse metabolic outcome, and subsequent post-hoc analyses are more prone to biases and type 2 error and should best be viewed as hypothesis-generating observations. Yet, the original hypothesis was clearly rejected, so MC accumulation, at least in VAT, is not associated with a worse metabolic phenotype. Regarding the estimation of MC accumulation, we did not use flow-cytometry methods that would have enabled characterizing MC sub-types, and potentially their activation state. Rather, we critically assessed the use of several, nicely intercorrelated, “MC-specific genes”, defining the AT-MC^low^ sub-group stringently, using combined thresholds for two genes (TPSB2 and CMA1) that would capture the two MC sub-populations. We also verified gene expression agreement with immunohistochemical assessment of AT-MC accumulation. Therefore, we cannot draw conclusions about MC activation state, including a possible association with LDL particles—which are known regulators of MC activity [21], although most of the associations we observed were between AT-MC accumulation and triglycerides, not with LDL or total cholesterol levels. Limiting the clinical implementation of our results is our use of VAT measures as a biomarker, since this AT is not accessible for sampling in the regular clinical setting. People with obesity have been shown to have higher levels of serum tryptase compared to lean people [6], and a positive association has been noted with BMI [22]. Yet, the source for serum tryptase is not restricted to MC in VAT, and therefore it is likely a poor correlate of visceral AT-MC accumulation. Therefore, future studies are required to assess whether MC-derived, circulating blood biomarkers, such as serum tryptase and/or chymase levels, could be clinically used to estimate visceral AT-MC accumulation and obesity sub-types. Our results suggest that clinically meaningful information may be obtained for those undergoing abdominal surgery for obesity management, by assessing tissues obtained during bariatric surgery molecularly and/or histopathologically. Although such analyses could be predictive of post-operative endpoints and may guide post-operative care, before obtaining more supportive evidence by other groups, it may still be premature to propose adipose tissue MC accumulation as a clinical predictor for the outcome of a bariatric surgery.

Although speculative, the data suggest putative mechanisms to explain the seemingly-positive effect of VAT-MC. Although our study was not focused on providing a systematic analysis of adipose tissue fibrosis, we report that in VAT, MC also populate fibrotic areas, and their gene expression correlates with several AT collagens (in SAT, correlations were only with COL6A1). Yet, recent studies suggest that in certain contexts, MC can exert anti-fibrotic/collagen-degrading effects that may result in a protective, rather than a pathogenic, role [23,24]. In addition, while AT fibrosis in mice is largely thought to contribute to tissue dysfunction [25,26], in humans, the pathological role of AT fibrosis may be more complex: higher SAT fibrosis may associate with poor metabolic and weight loss response to bariatric surgery [27], but VAT fibrosis associates with smaller adipocytes, potentially by limiting visceral adipocyte hypertrophy, thereby mediating better metabolic profile [28,29]. Additionally, MC secrete prostaglandins including 15-deoxy-delta PGJ2, which is an endogenous ligand for PPARγ, particularly in response to high-glucose conditions, resulting in increased adipogenesis [8]. Such MC-mediated PPARγ activation may support “healthy” AT expansion [30]. Indeed, the expression of CPA3, an MC marker, is associated with Uncoupling Protein 1 (UCP1) in the SAT of lean people, and MC-related histamine and IL-4 induce UCP1 expression in adipocytes, promoting AT beiging [31,32]. MC also contribute to angiogenesis, as demonstrated in cancer [33,34]. Thus, AT-MC adjacent to micro-vessels may imply their involvement in increased angiogenesis, possibly further supporting healthy AT expansion by limiting hypoxia. Finally, we show that AT-MC correlate with macrophages only in fibrotic areas, and at the whole-tissue level, MC genes may in fact inversely correlate with macrophage-specific genes or with macrophage CLS numbers. This suggests that higher MC accumulation in VAT might in fact associate with lower macrophage infiltration/activity, which in turn is indicative of a lower inflammatory burden-related adipose tissue dysfunction. Furthermore, MC^high^ exhibited greater postoperative weight loss. A putative explanation might be high preoperative fasting insulin that enabled greater postoperative decline in insulin levels, thereby contributing to more substantial weight loss.

## 5. Conclusions

Clinically used methods for better phenotyping people with obesity are still insufficient and greatly limit stratified or more precision obesity management. Our study suggests that VAT-MC accumulation estimates could be used as a tool for obesity sub-phenotyping. This can rely on relatively available laboratory procedures, such as molecular (real-time PCR) and/or histopathological assays using clinically common, known cell markers. Moreover, we propose that molecular and/or histological examinations of tissues obtained during surgery can uncover clinically important information, such as the prediction of post bariatric-surgery outcomes. This could aid in the post-operative management to optimize patient care in an individualized manner, similar to the common practice in the management of other diseases, such as cancer. Jointly, we propose that adipose tissue composition holds clinically relevant information that should be better exploited for improving the care provided to people with obesity.

## Figures and Tables

**Table 1:** Baseline characteristics of obese patient.

		Beer-Sheva (Main Cohort)			Leipzig-1 (Validation Cohort 1)			Leipzig 2 (Validation Cohort 2)		
	Normal Values/Units	Total	VAT-Low	VAT-High	Total	VAT-Low	VAT-High	Total	VAT-Low	VAT-High
N		**65**	19	46	**32**	7	25	**56**	18	38
Age	(year)	**46.7 ± 14.2**	50.8 ± 13.7	45.0 ± 14.3	**47.7 ± 15.3**	44.1 ± 8.5	48.9 ± 11.7	**43.9 ± 10.3**	45.4 ± 6.9	43.2 ± 11.6
Sex	M/F	**25/40**	8/11	17/29	**9/23**	1/6	8/17	**13/43**	3/15	10/28
Weight	(kg)	**112.5 ± 23.6**	111.7 ± 22.6	112.8 ± 24.2	**134.9 ± 32.6**	120.4 ± 24.3	139.0 ± 33.8	**144.2 ± 30.3**	132.2 ± 24.8	149.9 ± 31.2 *
BMI	<25 (kg/m^2^)	**40.7 ± 6.0**	40.2 ± 5.8	40.9 ± 6.2	**47.9 ± 11.2**	44.1 ± 8.5	48.9 ± 11.7	**50.5 ± 8.6**	47.9 ± 7.8	51.7 ± 8.8
FPG	3.9–5.4 mmol/L	**6.5 ± 2.7**	7.7 ± 3.9	6.0 ± 1.8	**6.7 ± 2.2**	6.8 ± 1.8	6.7 ± 2.3	**6.6 ± 1.7**	7.0 ± 1.8	6.4 ± 1.6
Insulin	<174 pmol/L	**105.1 ± 72.5**	89.9 ± 49.8	111.4 ± 79.8	**135.1 ± 158.9**	74.3 ± 49.5	147.2 ± 171.1	**206.1 ± 163.4**	211.6 ± 215.2	203.5 ± 135.1
HbA1c	<39 mmol/mol	**45.9 ± 16.7**	49.2 ± 22.6	43.9 ± 13.9	**44.3 ± 9.1**	47.7 ± 10.7	43.2 ± 8.5	**46.4 ± 11.8**	46.9 ± 9.6	46.2 ± 12.9
	<5.7%	**6.3 ± 1.6**	6.8 ± 2.1	6.2 ± 1.3	**6.2 ± 0.8**	6.5 ± 1.0	6.1 ± 0.8	**6.4 ± 1.1**	6.4 ± 0.9	6.4 ± 1.2
HOMA-IR	<2.5	**5.2 ± 3.8**	5.3 ± 3.8	5.1 ± 3.9	**6.7 ± 11.4**	3.0 ± 1.9	7.4 ± 12.3	**8.9 ± 7.4**	9.0 ± 9.3	8.8 ± 6.5
HOMA-β %		**173.4 ± 159.6**	112.4 ± 80.6	198.6 ± 177.4 *	**130.6 ± 92.3**	82.6 ± 66.7	140.3 ± 95.1	**219.3 ± 166.8**	210.6 ± 208.1	223.6 ± 145.9
Total cholesterol	<5.2 mmol/L	**4.8 ± 1.1**	5.1 ± 1.3	4.6 ± 1.0	**4.7 ± 1.1**	5.2 ± 1.2	4.4 ± 1.0	**5.3 ± 1.1**	5.2 ± 1.0	5.3 ± 1.2
LDL-c	<1.8 mmol/L	**2.8 ± 0.9**	2.7 ± 0.9	2.8 ± 0.9	**3.2 ± 1.3**	3.3 ± 0.9	3.2 ± 1.5	**3.3 ± 1.0**	3.1 ± 0.5	3.4 ± 1.2
Triglycerides	<1.7 mmol/L	**2.0 ± 1.2**	2.7 ± 1.6	1.7 ± 0.8 *	**1.9 ± 1.0**	1.7 ± 0.8	1.9 ± 1.2	**2.0 ± 1.0**	1.9 ± 0.7	2.1 ± 1.1
HDL: male	>1.03 mmol/L	**1.0 ± 0.2**	1.0 ± 0.2	1.0 ± 0.2	**1.1 ± 0.2**	1.2 ± 0.2	1.1 ± 0.2	**1.1 ± 0.3**	1.0 ± 0.1	1.1 ± 0.4
female	>1.29 mmol/L	**1.2 ± 0.4**	1.2 ± 0.4	1.2 ± 0.4				**1.2 ± 0.3**	1.2 ± 0.4	1.2 ± 0.3
TG/HDL ratio: male		**2.6 ± 1.5**	3.1 ± 1.8	2.3 ± 1.4	**1.8 ± 1.2**	1.3 ± 0.6	2.0 ± 1.3	**2.6 ± 1.9**	2.0 ± 0.4	2.7 ± 2.1
female		**1.6 ± 1.3**	2.2 ± 2.1	1.4 ± 0.8 *				**1.7 ± 1.1**	1.6 ± 0.9	1.7 ± 1.2
CRP	<47.6 nmol/L	**15.7 ± 25.2**	10.5 ± 9.7	17.9 ± 29.2	**17.6 ± 19.1**	10.1 ± 8.6	19.4 ± 20.6	**59.4 ± 65.3**	46.5 ± 63.8	65.4 ± 65.6
AST	<0.68 µkat/L	**0.5 ± 0.4**	0.6 ± 0.4	0.5 ± 0.4	**0.7 ± 0.7**	0.5 ± 0.1	0.8 ± 0.8	**0.6 ± 0.3**	0.6 ± 0.3	0.6 ± 0.3
ALT	<0.68 µkat/L	**0.6 ± 0.4**	0.7 ± 0.4	0.5 ± 0.4	**0.7 ± 0.5**	0.5 ± 0.1	0.8 ± 0.6	**0.7 ± 0.4**	0.7 ± 0.5	0.6 ± 0.4
AP	<2 µkat/L	**1.5 ± 0.5**	1.8 ± 0.6	1.4 ± 0.4 *						
Diastolic BP	<85 mmHg	**81.0 ± 17.2**	88.2 ± 27.7	78.4 ± 10.6						
Systolic BP	<130 mmHg	**138.5 ± 16.1**	139.1 ± 19.7	138.3 ± 14.8						
Visceral Adipocyte area	(µm^2^)	**4213.6 ± 1323.8**	4455.8 ± 1138.7	4097.8 ± 1412.8						
Subcutaneous Adipocyte area	(µm^2^)	**5720.9 ± 1284.1 ^†^**	5771.7 ± 1337.8	5696.7 ± 1290.7						
SAT-KIT		**1.0 ± 0.7**	0.8 ± 0.3	1.1 ± 0.8	**5.9 ± 0.3**	5.9 ± 0.2	5.9 ± 0.4	1.2 ± 0.7	1.0 ± 0.5	1.3 ± 0.8
SAT-TPSB2		**1.2 ± 0.8**	1.23 ± 0.8	1.2 ± 0.8	**6.3 ± 0.5**	6.1 ± 0.3	6.2 ± 0.5	1.2 ± 0.9	0.8 ± 0.5	1.3 ± 0.9 *
SAT-CMA1		**2.1 ± 1.0**	1.6 ± 0.8	2.3 ± 1.1	**5.3 ± 0.1**	5.2 ± 0.1	5.3 ± 0.5	1.3 ± 0.8	1.1 ± 1.1	1.3 ± 0.6
VAT fibrosis grade		**1.8 ± 0.7**	1.8 ± 0.6	1.8 ± 0.7						

Clinical characteristics of participants with obesity from the Beer–Sheva and Leipzig cohorts included in this study. VAT-MC^low/high^ were defined based on VAT expression of CMA1 and TPSB2, as detailed in methods (see also Table S2 for MC^low/high^ definition based on VAT expression of KIT). M, male; F, female; FPG, fasting plasma glucose; HOMA-IR, homeostatic model assessment of insulin resistance; HOMA-β, homeostatic model assessment of beta cells reserve; LDL-c, low-density lipoprotein cholesterol; TG, triglycerides; HDL-c, high-density lipoprotein cholesterol; CRP, c-reactive protein; AST; aspartate aminotransferase; ALT, alanine transaminase; AP, alkaline phosphatase; VAT-MC: visceral adipose tissue with mast cells; Values are mean ± standard deviation. * *p* < 0.05 different from VAT-MC^low^ by independent *t*-test. ^†^ *p* < 0.05 compared visceral adipocyte area, by paired *t*-test. ^#^ C- reactive protein (CRP) values are higher compared with validation cohort 1 and the main cohort since inclusion criterion of patients with CRP < 47.6 nmol/L (5.0 mg/L) was not applied in this cohort.

**Table 2:** Intercorrelation between VAT-MC and collagens’ gene expression in the Beer-Sheva cohort (*n* = 65). n.s, not significant. Values are *r* and *p* value (upper and lower line respectively) of Spearman’s correlations. CMA1: mast cell chymase 1, TPSB2: mast cell tryptase beta II, COLA1A1: collagen type I alpha 1 chain, COLA3A1: collagen type III alpha 1 chain, COLA6A1: collagen type VI alpha 1 chain.

	KIT	TPSB2	CMA1	COLA3A1	COLA6A1
COLA1A1	0.342	n.s.	0.236	0.895	0.305
	0.007		0.065	<0.001	0.015
COLA3A1	0.333	n.s.	0.275		0.303
	0.009		0.032		0.017
COLA6A1	n.s.	0.435	0.26		
		<0.001	0.038		

**Table 3:** Changes in clinical and inflammatory markers and AT genes, 12 months following bariatric surgery in validation cohort 2. Values are mean ± SD. CLS—crown-like structures, VAT—visceral adipose tissue, SAT—subcutaneous adipose tissue. ns—not significant (*p* > 0.05).

Characteristics	Baseline (*n* = 56)	After 12 Months (*n* = 56)	*p*-Value
Weight (kg)	144.2 ± 30.3	105.6 ± 23.8	<0.0001
BMI (kg/m^2^)	50.5 ± 8.6	37.1 ± 8.0	<0.0001
Body fat (%)	45.1 ± 7.6	35.5 ± 8.2	<0.0001
FPG (mmol/L)	6.6 ± 1.7	5.6 ± 1.5	<0.0001
HbA1c (%)	6.4 ± 1.1	5.4 ± 0.8	<0.0001
Insulin (pmol/L)	206.1 ± 163.4	88.8 ± 102.4	<0.0001
HOMA-IR	8.9 ± 7.4	3.8 ± 5.3	<0.0001
Total cholesterol (mmol/L)	5.3 ± 1.1	4.9 ± 0.9	0.067
LDL-c (mmol/L)	3.3 ± 1.0	3.1 ± 1.0	ns
HDL (mmol/L)	1.2 ± 0.3	1.4 ± 0.4	<0.0001
Triglycerides (mmol/L)	2.0 ± 1.0	1.3 ± 0.6	<0.0001
CRP (nmol/L)	59.4 ± 65.0	32.9 ± 42.8	0.024
Interleukin 6 (pg/mL)	5.5 ± 3.4	2.6 ± 2.0	<0.0001
Interleukin 1 beta (pg/mL)	9.6 ± 6.7	9.1 ± 8.0	ns
Tumor necrosis factor alpha (pg/mL)	8.3 ± 3.1	8.0 ± 3.7	ns
**VAT**			
CLS (per 100 adipocytes)	9.0 ± 2.9	7.7 ± 3.3	<0.0001
*TNF* (AU)	2.3 ± 1.9	1.7 ± 1.9	ns
*IL1b* (AU)	1.9 ± 1.2	1.2 ± 0.9	0.003
*TGFb* (AU)	1.5 ± 1.4	1.4 ± 1.1	ns
*KIT* (AU)	1.1 ± 0.7	1.1 ± 0.7	ns
*TPSB2* (AU)	1.0 ± 1.0	1.1 ± 1.0	ns
*CMA1* (AU)	1.2 ± 1.1	0.8 ± 1.0	0.044
**SAT**			
CLS (per 100 adipocytes)	4.3 ± 2.1	3.6 ± 2.3	0.005
*TNF* (AU)	1.9 ± 1.8	1.5 ± 1.2	ns
*IL1b* (AU)	1.9 ± 1.8	1.3 ± 0.9	0.035
*TGFb* (AU)	1.5 ± 0.9	1.2 ± 0.7	0.024
*KIT* (AU)	1.3 ± 0.7	1.1 ± 0.8	ns
*TPSB2* (AU)	1.2 ± 0.9	1.1 ± 0.7	ns
*CMA1* (AU)	1.3 ± 0.8	1.4 ± 1.0	0.146

## Supplementary Materials

The following are available online at https://www.mdpi.com/2073-4409/9/6/1508/s1, Figure S1: The expression of KIT in cells that can be found in human AT, Figure S2: The expression of TPSB2 in cells that can be found in human AT, Figure S3: The expression of CMA1 in cells that can be found in human AT, Figure S4: SAT C-Kit+ cells within fibrotic area and fibrosis grading. Figure S5: Comparison of clinical parameters between participants with high versus low MC accumulation in VAT, stratified by sex or obesity category. Table S1: PCR probe ID. Table S2: Clinical characteristics of participants with obesity from the Beer–Sheva and Leipzig cohorts, each also stratified by VAT-MC low/high based on expression of KIT. Table S3: Inter-correlation between SAT-MC gene expression and SAT-collagens. Table S4: Usage of medications in persons with VAT MC^high^ and MC^low^ among the entire main cohort. Table S5: Diabetes duration and medication among persons with type 2 diabetes stratified to VAT MC^high^ and MC^low^, main cohort. Table S6: Association between SAT-MC genes expression and clinical parameters in the three cohorts.
