# Supplementary material for: Higher Mast Cell Accumulation in Human Adipose Tissues Defines Clinically Favorable Obesity Sub-Phenotypes
Source: Cells. 2020 Jun 20;9(6):1508. doi: 10.3390/cells9061508 (PMC7349306; doi:10.3390/cells9061508)
Supplement: Supplementary file 1 [file cells-09-01508-s001.pdf]

Supplemental Figures

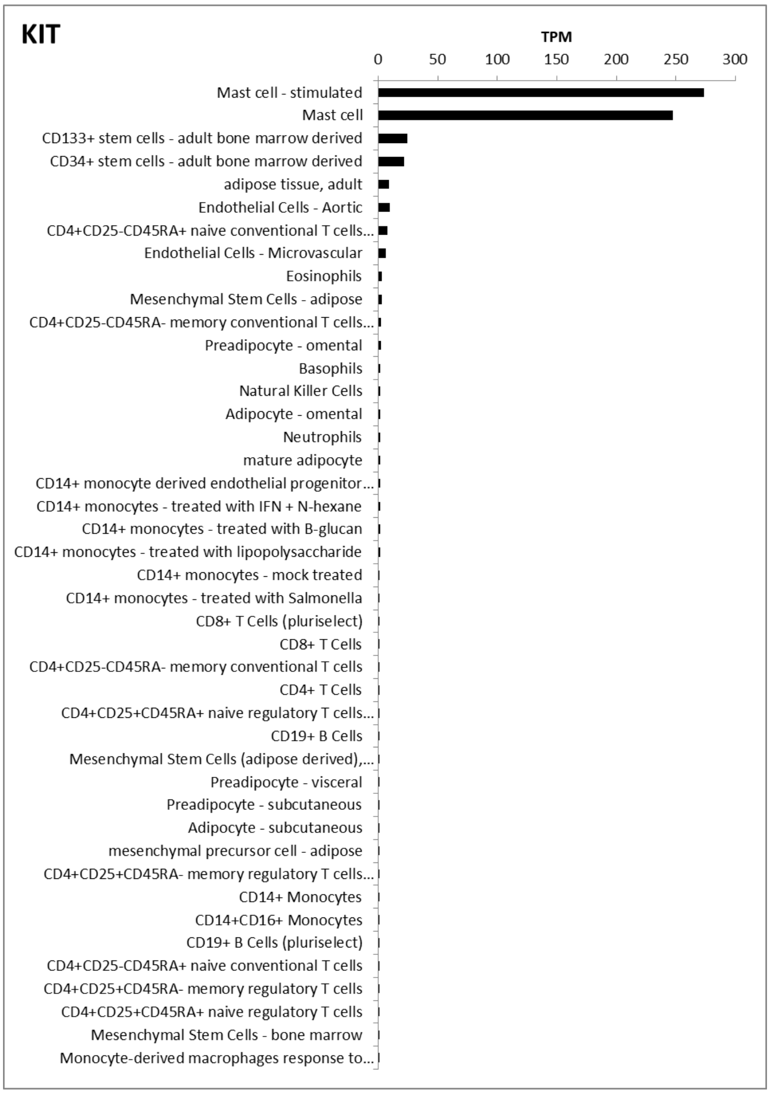

Figure S1: The expression of KIT in cells that can be found in human AT.

We mined a published human gene expression dataset of 556 tissues, cells, and cell lines (Ono H, *et al.,* RefEx, a reference gene expression dataset as a web tool for the functional analysis of genes. *Sci Data*. **2017**;4:170105). KIT was found to be most highly expressed in Mast Cells (MC) by at least 11-fold compared to the next highest KIT expressing cell population (hematopoietic stem cells). (TPM, tags per million).

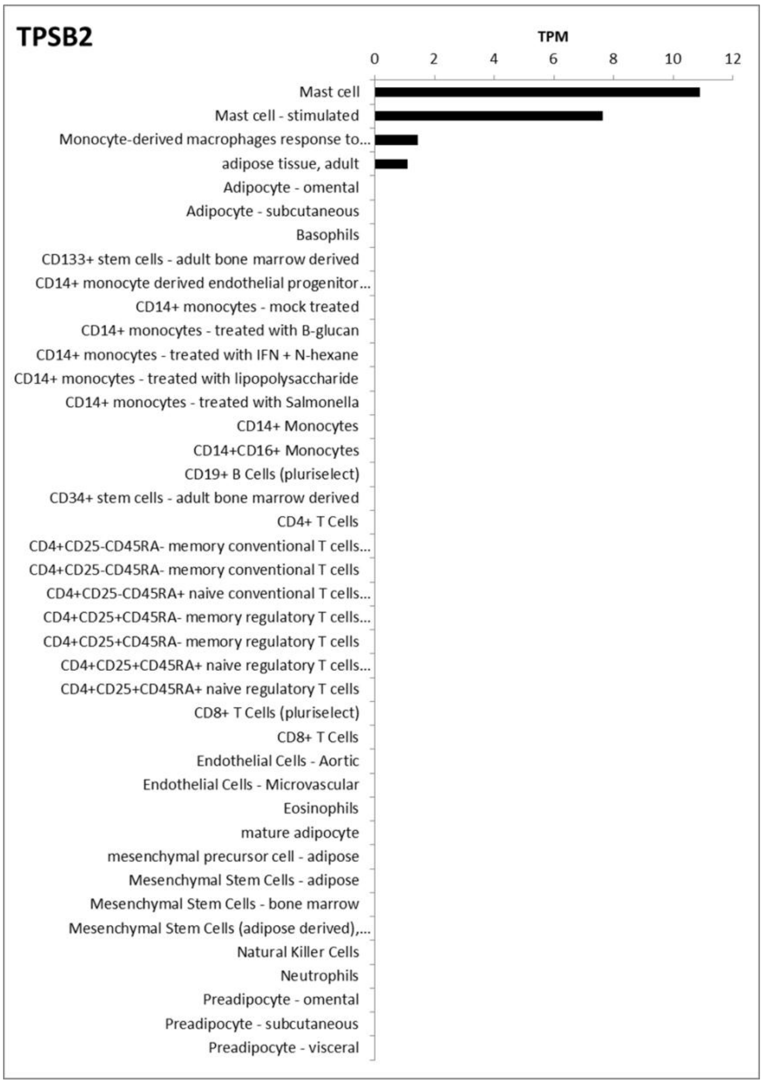

Figure S2: The expression of TPSB2 in cells that can be found in human AT.

We mined a published human gene expression dataset of 556 tissues, cells, and cell lines (Ono H, *et al.,* RefEx, a reference gene expression dataset as a web tool for the functional analysis of genes. *Sci Data*. **2017**;4:170105). TPSB2 was found to be ~7-fold more highly expressed than the next-highest expressing, influenza-stimulated monocyte-derived macrophages.

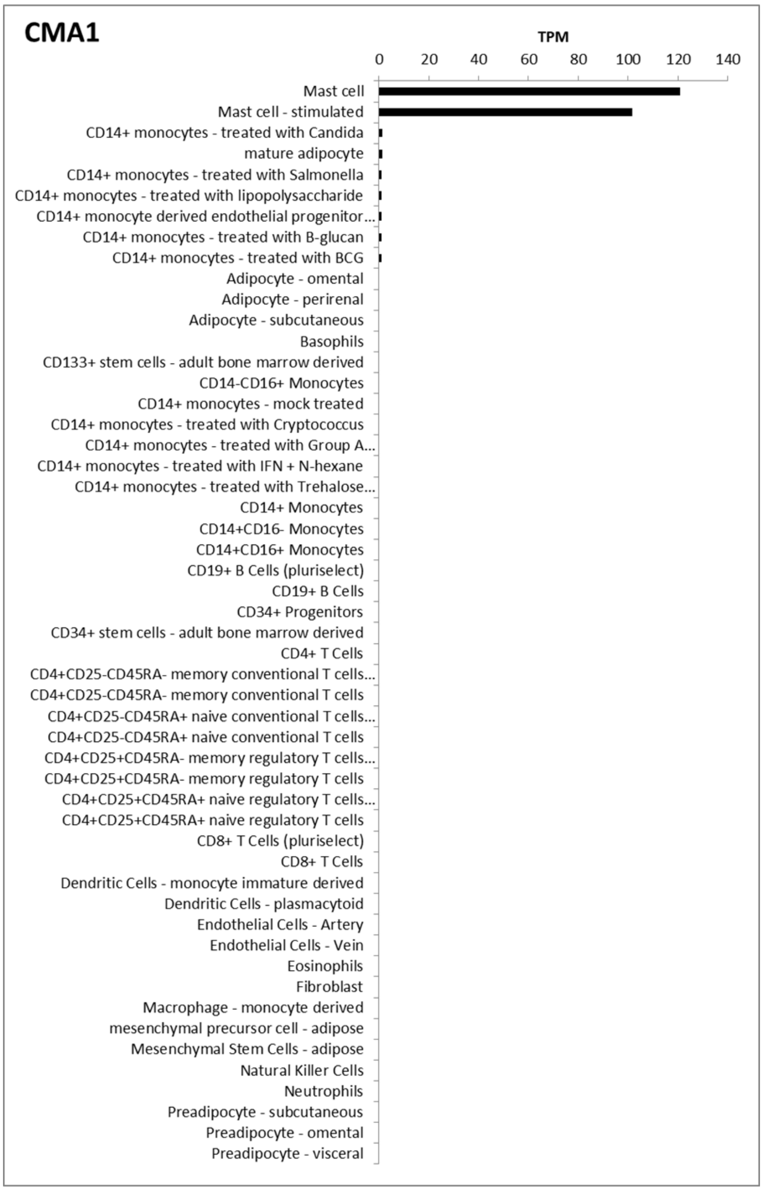

Figure S3: The expression of CMA1 in cells that can be found in human AT.

We mined a published human gene expression dataset of 556 tissues, cells, and cell lines (Ono H, *et al.,* RefEx, a reference gene expression dataset as a web tool for the functional analysis of genes. *Sci Data*. **2017**;4:170105). CMA1 was most specific to MC, being 86-fold more highly expressed than candida-treated monocytes

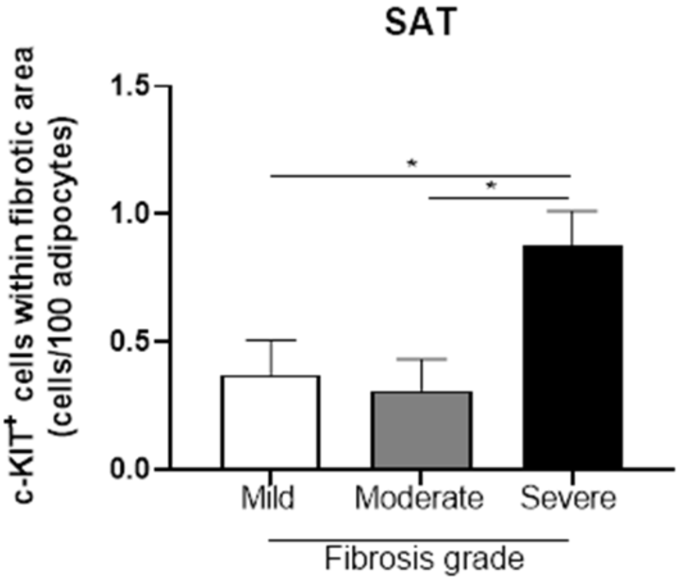

Figure S4: SAT C-Kit+ cells within fibrotic area and fibrosis grading. Numbers of SAT C-Kit+ cells in sections rated by clinical pathologists (co-authors YD and RSL) as exhibiting mild, moderate or severe degree of fibrosis (n=31). *$p<0.05$

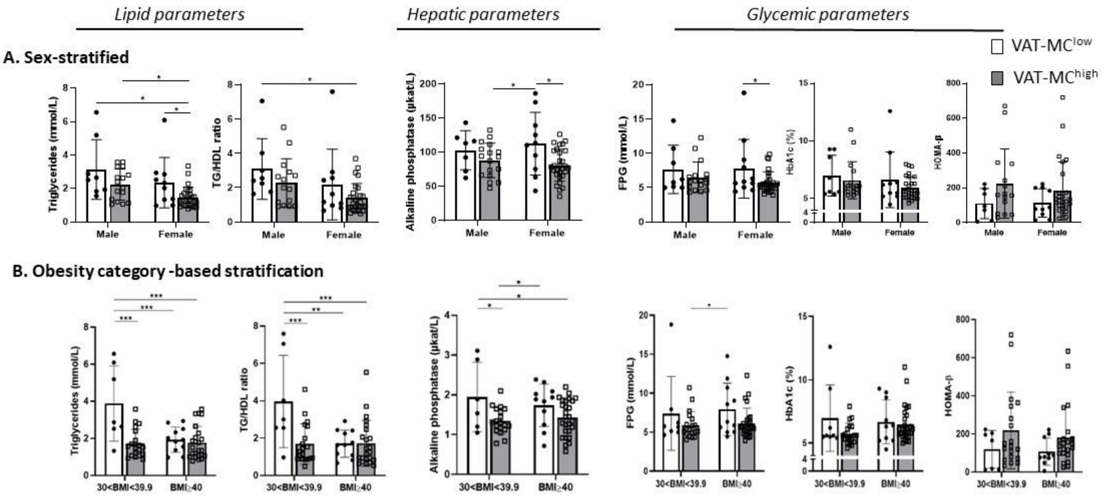

Figure S5: Comparison of clinical parameters between participants with high versus low MC infiltration in VAT, stratified by sex or obesity category. Participants from Beer-Sheva cohort (cohort 1) were stratified based on sex **(A)** or obesity category A-B (30≤BMI<40 kg/m^2) or class C (BMI≥40 kg/m^2). Differences in triglycerides, TG/HDL ratio, Alkaline phosphatase, FPG, HbA1c and HOMA-β between VAT- MC^{low} versus VAT- MC^{high}. Age-adjusted LSD post-hoc test was used to compare between groups. Values are expressed as mean ± SD. *p<0.05, **p<0.01, ***p<0.001.

Supplemental Tables

Table S1: PCR probe ID

	**Gene**	**Probe ID**
1.	KIT	Hs00174029_m1
2.	TPSB2	Hs02576518_gH
3.	CMA1	Hs01095979_g1
4.	PGK1	Hs00943178_g1
5.	PPIA	Hs99999904_m1

Table S2: **Clinical characteristics of participants with obesity from the Be’er-Sheva and Leipzig cohorts, each also stratified by VAT-MC low/high based on expression of KIT**

	Beer-Sheva (main cohort)			Leipzig-1 (validation cohort 1)			Leipzig 2 (validation cohort 2)		
	**total**	**VAT-KIT^{low}**	**VAT-KIT^{high}**	**total**	**VAT-KIT^{low}**	**VAT-KIT^{high}**	**total**	**VAT-KIT^{low}**	**VAT-KIT^{high}**
N	65	32	32	32	16	16	56	24	23
Age (year)	46.7±14.2	49.0±15.2	45.0±13.1	47.7±15.3	54.0±16.0	41.4±12.1*	43.9±10.3	47.4±10.1	40.6±9.3*
Sex M/F	25/40	13/19	12/20	9/23	6/10	3/13	13/43	7/17	5/18
Weight (kg)	112.5±23.6	111.5±22.3	113.6±25.4	134.9±32.6	122.3±28.7	147.6±32.1*	144.2±30.3	139.8±28.5	148.1±28.6
BMI (kg/m^2)	40.7±6.0	39.6±5.4	41.7±6.6	47.9±11.2	44.2±11.4	51.6±10.0	50.5±8.6	47.9±7.4	52.5±8.3
FPG (mmol/L)	6.5±2.7	6.8±3.3	6.3±1.9	6.7±2.2	7.0±2.2	6.3±2.2	6.6±1.7	7.1±1.7	6.0±1.4*
Insulin (nmol/L)	105.1±72.5	101.8±78.7	108.6±66.6	135.1±158.9	89.1±64.4	168.0±197.6	206.1±163.4	212.5±134.2	186.8±124.2
HbA1c (mmol/mol)	45.9±16.7	45.8±18.5	45.2±15.3	44.3±9.1	44.8±9.7	43.7±8.8	46.4±11.8	47.2±10.3	46.8±13.4
(%)	6.3±1.6	6.4±1.7	6.3±1.4	6.2±0.8	6.2±0.9	6.1±0.8	6.4±1.1	6.5±0.9	6.4±1.2
HOMA-IR	5.2±3.8	5.0±3.8	5.3±3.9	6.7±11.4	3.8±2.7	8.8±14.6	8.9±7.4	9.8±7.0	7.3±4.7
HOMA-β %	173.4±159.6	173.1±172.7	173.6±147.5	130.6±92.3	93.5±82.3	157.2±92.6	219.3±166.8	189.4±126.0	238.4±154.8
Total cholesterol (mmol/L)	4.8±1.1	4.9±3.8	4.7±1.1	4.7±1.1	5.0±1.1	4.3±1.0	5.3±1.1	5.4±1.3	5.0±1.0
LDL-c (mmol/L)	2.8±0.9	2.7±0.8	2.9±1.0	3.2±1.3	3.3±1.2	3.2±1.5	3.3±1.0	3.5±1.0	3.2±1.1
TG (mmol/L)	2.0±1.2	2.3±1.4	1.8±0.8	1.9±1.0	2.0±1.1	1.7±1.0	2.0±1.0	2.3±1.1	1.6±0.7*
HDL (mmol/L): male	1.0±0.2	1.0±0.2	1.1±0.2	1.1±0.2	1.0±0.1	1.2±0.2	1.1±0.3	1.1±0.4	1.1±0.2
female	1.2±0.4	1.3±0.3	1.2±0.4				1.2±0.3	1.3±0.3	1.1±0.3
TG/HDL ratio: male	2.6±1.5	2.8±1.5	2.2±1.6	1.8±1.2	2.0±1.3	1.5±1.1	2.6±1.9	2.5±1.6	1.9±1.5
female	1.6±1.3	1.7±1.6	1.5±0.9				1.7±1.1	2.0±1.4	1.4±0.7
CRP (nmol/L)	15.7±25.2	16.3±32.0	15.0±15.8	17.6±19.1	23.4±25.2	12.1±8.5	59.4±65.3	66.0±73.1	57.1±59.7
AST (μkat/L)	0.5±0.4	0.5±0.2	0.6±0.5	0.7±0.7	0.8±1.0	0.6±0.2	0.6±0.3	0.6±0.3	0.6±0.4
ALT (μkat/L)	0.6±0.4	0.5±0.3	0.6±0.3	0.7±0.5	0.6±0.5	0.8±0.5	0.7±0.4	0.7±0.5	0.7±0.3
AP (μkat/L)	1.5±0.5	1.5±0.6	1.5±0.4						
Diastolic BP (mmHg)	81.0±17.2	83.1±23.4	79.3±10.8						
Systolic BP (mmHg)	138.5±16.1	140.4±19.5	137.2±13.4						
Visceral Median Adipocyte area (μm^2)	4213.6±1323.8	4170.8±1372.9	4203.4±1344.5						
Subcutaneous Median Adipocyte area (μm^2)	5720.9±1284.1 †	5882.4±930.0	5610.4±1551.0						
SAT-KIT	1.0±0.7	0.8±0.5	1.2±0.8	5.9±0.3	5.9±0.4	5.9±0.2	1.2±0.7	1.0±0.6	1.4±0.7
SAT-TPSB2	1.2±0.8	1.3±1.0	1.2±0.7	6.3±0.5	6.1±0.3	5.3±0.1	1.2±0.9	1.3±0.9	1.2±0.8
SAT-CMA1	2.1±1.0	1.7±1.0	2.4±1.0	5.3±0.1	5.3±0.3	6.2±0.5	1.3±0.8	1.2±1.0	1.3±0.8
VAT fibrosis grade	1.8±0.7	1.7±0.6	1.8±0.7						

VAT-MC accumulation was stratified based on the expression of VAT-KIT (below/above median). M, male; F, female; FPG, fasting plasma glucose; HOMA-IR, homeostatic model assessment of insulin resistance; HOMA-β, homeostatic model assessment of beta cells reserve; LDL-c, low density lipoprotein cholesterol; TG, triglycerides; HDL-c, high density lipoprotein cholesterol; CRP, c-reactive protein; AST; aspartate aminotransferase; ALT, alanine transaminase; AP, alkaline phosphatase; Values are mean ± standard deviation. * $p<0.05$ different from VAT-KIT^{low} by independent t-test. † $p<0.05$ compared visceral adipocyte area, by paired t-test.

Table S3: Inter-correlation between SAT-MC gene expression and SAT-collagens

	**KIT**	**TPSB2**	**CMA1**	**COLA3A1**	**COLA6A1**
**COLA1A1**	n.s	n.s.	n.s.	**0.544**<br>**<0.001**	**0.321**<br>**0.050**
**COLA3A1**	n.s	n.s.	n.s		n.s
**COLA6A1**	**0.356**<br>**0.028**	**0.564**<br>**<0.001**	**0.342**<br>**0.036**		

Inter-correlation between SAT-MC and collagens gene expression in the Beer-Sheva main cohort (n=39). n.s, not significant. Values are r and p value (upper and lower line, respectively) of Spearman's correlations.

Table S4: Usage of medications in persons with VAT MC^{high} and MC^{low} among the entire main cohort

Entire cohort				
Medications for:		Low (n=19)	High (n=46)	
Diabetes:	Insulin	5.3%	8.7%	ns
	Non-insulin	36.8%	28.3%	ns
Statins		15.8%	17.4%	ns
BP		21.1%	23.9%	ns
Fibrate		5.3%	4.3%	ns
Hypo-thyroid		10.5%	4.3%	ns
Asthma		0.0%	8.7%	ns
Aspirin		21.1%	8.9%	ns
Reflux		5.3%	13.3%	ns

Table S5: Diabetes duration and medication among persons with type 2 diabetes stratified to VAT MC^{high} and MC^{low}, main cohort.

Diabetes only				
Medications for:		Low (n=9)	High (n=16)	
Diabetes:	Insulin	11.1%	25.0%	ns
	Non-insulin	77.8%	81.3%	ns
Statins		22.2%	12.5%	ns
BP		33.3%	43.8%	ns
Fibrate		11.1%	12.5%	ns
Hypo-thyroid		11.1%	0.0%	ns
Asthma		0.0%	6.3%	ns
Aspirin		44.4%	26.7%	ns
Reflux		11.1%	20.0%	ns
Diabetes duration (months)		55.2±66.1	80.2±71.8	ns

Table S6: Association between SAT-MC genes expression and clinical parameters in the three cohorts.

	Beer-Sheva (main cohort)			Leipzig-1 (Validation cohort 1)				Leipzig-2 (Validation cohort 2)		
	KIT	CMA1	TPSB2	KIT 1	KIT 2	CMA1	TPSB2	KIT	CMA1	TPSB2
Age	0.054	-0.037	0.264	-0.352*	-0.211	-0.103	0.155	-0.241	0.137	-0.112
Weight	-0.435**	-0.367*	-0.559**	0.153	0.063	0.197	-0.186	0.090	0.322*	0.007
BMI	-0.109	0.036	-0.371*	0.190	0.123	0.191	-0.225	0.228	0.192	0.047
FPG	0.003	-0.013	0.152	-0.275	-0.230	-0.291	0.323	-0.209	0.005	-0.082
Insulin	-0.213	-0.127	-0.172	-0.177	-0.178	-0.161	0.172	0.102	0.275	-0.020
HbA1c	-0.018	-0.047	0.002	-0.331	-0.138	-0.310	-0.045	-0.063	-0.022	-0.092
HOMA-IR	-0.173	-0.116	-0.076	-0.162	-0.190	-0.225	0.209	0.041	0.241	-0.083
HOMA-ß %	-0.068	0.024	-0.115	-0.057	-0.043	-0.009	0.131	0.256	0.281*	0.039
Total cholesterol	0.153	0.038	0.053	0.069	0.054	-0.429	-0.039	0.186	-0.175	0.105
LDL-c	0.196	0.187	-0.022	0.019	0.119	-0.393	0.142	0.119	0.135	0.254
TG	0.017	0.006	0.140	-0.147	-0.208	-0.340	0.016	-0.017	-0.212	0.127
HDL	0.105	-0.004	0.021	0.492*	0.533*	-0.279	-0.163	-0.048	-0.004	-0.036
TG/HDL ratio	-0.068	-0.021	0.061	-0.500	-0.518*	-0.114	-0.096	-0.009	-0.172	0.056
CRP	0.120	0.163	-0.113	0.195	0.234	-0.003	0.214	-0.095	-0.012	-0.103
AST	-0.018	-0.006	-0.013	0.081	0.003	0.211	-0.054	0.144	0.065	-0.018
ALT	-0.101	-0.087	-0.236	-0.095	-0.264	-0.005	-0.235	0.087	-0.058	-0.086
AP	-0.284	-0.300	-0.124							
Diastolic BP	-0.268	-0.184	-0.239							
Systolic BP	-0.258	-0.221	-0.284							

Color scale: 0.5 / 0 / -0.5

Spearman's correlation between MC genes expression and clinical parameters in three independent cohorts. Values are spearman's correlation coefficient. Red and green colors express negative and positive associations, respectively. *p<0.05; **p<0.01.
